# University of Buffalo, 1846-1911

**Published:** 1911-07

**Authors:** 


### University of Buffalo, 1846-1911

The commencement exercises of the University of Buffalo were held at the Teck Theater on June 1 and the four departments graduated 153 students divided as follows: Medicine 41; pharmacy 50; law 35; dentistry 27. There were eight women in the classes, four in medicine, two in pharmacy and one each in law and dentistry.

The theater was filled to its capacity and was handsomely decorated with American colors and the University blue and white. Alma Mater was sung after which Dr. Charles A. Jessup offered prayer. Dr. Eli H. Long presented the graduates in medicine to Chancellor Charles P. Norton who conferred the degrees. The Hippocratic oath was administered by Dr. Matthew D. Mann. Diplomas were given the graduates of the other departments, the class in pharmacy being presented by Dr. E. J. Kiepe; in law by George D. Crofts and in dentistry by Dean George B. Snow.

The exercises were closed by an address to the graduates by Professor Herman L. Fairchild of the University of Rochester.

The class in medicine follows:

Egbert Lamonte Burhyte, James Jay Clark, Elmer A. D. Clarke, Albert Jackson Colcord, Clarence Vincent Costello, James Frank Crawford, A.B.; Charles Milton Denison, Rose Rudolph Donk, A.B.; Frances Mabel Flood, Edward Peter Forrestel, Eva Grace Fowler, Guido James Gianfranceshi, Charles Handel, Morris Eby Heck, Marshall Ligon Hillsman, A. B.; Lucy Alice Kenner, Charles Arthur Lawler, Charles Leone, Hyman Loeb Levin, Mansfield Goldberg Levy, Hugh Chauncey McDowell, Walter Lewis Machemer, Nathalie Kavaleff Mankell, Herbert Christian Mann, Harry R. Marlatt, Edward Henry Mehl, George Leslie Miller, Arthur Allan Mitten, Albert Frederick Ostwald, Augustus Charles Paul, Leon Hastings Prior, Charles Carroll Ransom, Wellington Manely Ross, Arthur Leonard Runals, Paul Henry Sandreczki, Anthony Costanza Scinta, James Edward Short, George Edward Slotkin, Paul Bryant Stewart, John Waugh Tong, Leon Mitchell Wilbor.

The two days preceding the commencement exercises were busy ones for the visiting alumni. There were class reunions, chief of which was that of the class of 1901, receptions, luncheons, and fraternity meetings. An entire day was given up to clinics at the hospitals in which the teaching staff of the medical department of the University took part and every department of medicine and surgery was liberally represented. The alumni association of the medical department elected the following named officers at the annual meeting:

President, Charles L. Preisch, of Lockport; vice-presidents, Nellie V. Chappell, of Buffalo; Eugene B. Horton, of Niagara Falls; E. A. Forsyth, of Buffalo; Jacob E. K. Morris, of Olean and B. F. Rogers, of Buffalo; secretary, D. K. McKenney, of Buffalo; treasurer, W. F. Jacobs of Buffalo; trustee for five years, Marshall Clinton of Buffalo; executive committee, L. Kauffman, Chester C. Cott and William P. Getman of Buffalo.

The faculty of the medical department gave a luncheon for the graduates at the University Club following the commencement exercises.

The reunion of the class of 1901 was a busy affair from the time the out-of-towners began to arrive until the end of the celebration dinner at the Statler hotel early in the morning of June 1.

There were 45 members of this class and more than half were gathered together at the luncheon given May 31. In the afternoon the class was given a sea voyage to Crystal Beach and return as an appetizer for the star event, the dinner of the first class reunion of the class since the rosy day of a decade ago when the members were displayed in cap and gown and received their diplomas.

There were eighteen members present at the dinner which began at the hour usual for such occasions and ended only when the last bit of canned oratory had been released from cold storage. Then the 1901ers clasped hands and sang Auld Lang Syne and that was all.

It was a great reunion and a great dinner and most enjoyable. Every man present at the gathering supplied his share of entertainment with song, story or music and nearly all the individual speeches were recitals of personal experiences during the ten years since graduation. These were pages from life and they ran the gamut of nearly every emotion known to imaginative literature and experienced humanity save that rare thrill which comes with the discovery that one has a callous on the outside of one thumb where the scissors bind as their blades cut through that heavy bank paper on which coupons are printed. There were tales of trials and early failures which struck a responsive chord in every heart; there were recitals of successes in the struggle with disease and death and there were incidents of quaint and humorous turn. It was wholly delightful, wholly personal and wholly reminiscent and human.

The president of the class, Dr. Ira P. Trevett read letters of regret from many absent members and spoke feelingly of the only vacancy caused by death, Dr. M. Elizabeth Shugens.

Among the out of town members present were: H. M. Burritt of Hilton; William W. Carlton of Waterloo; George H. Davis of LeRoy; B. F. Illston of Jamestown; John F. Kane of Olean; Paul O. Luedeke of Rochester; Ira P. Trevett of Lackawanna; Eli H. Vail of Churchville; John A. Weidman of Dunkirk; Lee A. Whitney of Rochester and John W. Riley of Oklahoma City, Okla.

The annual banquet of the alumni of the department of pharmacy was held at the Statler hotel on June 1 with 125 guests present. The feature of the pharmacy celebration was the presentation by the alumni association to Dr. Willis G. Gregory of a handsome gold watch in commemoration of the completion of his 25th year as professor of pharmacy in the university.

